# A comparative analysis of anthropometric indices for identifying prediabetes in US adults: results from NHANES 2021–2023

**DOI:** 10.1016/j.pmedr.2025.103345

**Published:** 2025-12-08

**Authors:** Xi Chen, Lijun Yan, Jie Yang, Shufang Yang

**Affiliations:** a Department of Endocrinology, The Affiliated Taizhou People's Hospital of Nanjing Medical University, Taizhou School of Clinical Medicine, Nanjing Medical University, Taizhou, Jiangsu, China; b Department of Geriatric Gastroenterology, The First Affiliated Hospital with Nanjing Medical University, Nanjing Medical University, Nanjing, China

**Keywords:** Prediabetes, Anthropometry, Body mass index, NHANES, Diagnosis

## Abstract

**Objective:**

This study evaluates the association between various anthropometric indices and prediabetes prevalence among adults in the United States.

**Methods:**

We conducted a cross-sectional analysis on participants aged ≥20 years using data from the National Health and Nutrition Examination Survey (NHANES) cycles 2021–2023. Weight, height, waist circumference, and hip circumference were used to calculate indices such as BMI, waist-to-hip ratio (WHR), waist-to-height ratio (WHtR), conicity index, abdominal volume index (AVI), body roundness index (BRI), body adiposity index (BAI), and a body shape index (ABSI). Multivariable logistic regression assessed associations between indices and prediabetes risk. Receiver Operating Characteristic (ROC) curve analysis evaluated the discriminatory power of each index.

**Results:**

Among 2515 participants, 49.6 % had prediabetes. Higher quartiles of BMI, Waist circumference, WHR, WHtR, CI, AVI, BRI, BAI, and ABSI were significantly associated with increased prediabetes risk (*P* < 0.05). WHR demonstrated the highest discriminatory power (AUC: 0.695; 95 % CI; 0.674, 0.716), followed by conicity index (AUC; 0.693; 95 % CI; 0.672, 0.714).

**Conclusions:**

Central adiposity measures, particularly WHR and CI, are more effective than BMI in identifying individuals at risk for prediabetes. However, due to the cross-sectional nature of our study, causal relationships between anthropometric indices and prediabetes risk cannot be established.

## Highlights

• Waist-to-hip ratio showed the strongest association with prediabetes.
• Central adiposity measures, such as WHR and conicity index, best predicted prediabetes.
• BMI showed weak discrimination, while the body adiposity index performed poorest.

## 1 Introduction

Prediabetes is a condition where blood sugar levels are higher than normal but not yet high enough to be classified as diabetes. The American Diabetes Association (ADA) has established specific criteria for diagnosing prediabetes in 2025. The diagnostic criteria for prediabetes include a fasting blood glucose level (FBG) between 100 and 125 mg/dL (5.6 to 6.9 mmol/L) or a blood glucose level between 140 and 199 mg/dL (7.8 to 11.0 mmol/L) two hours after consuming a 75-g glucose solution or an HbA1c level between 5.7 % and 6.4 % (Committee, 2024). This state increases the risk of developing type 2 diabetes, heart disease, and stroke. In 2019, the Centers for Disease Control and Prevention (CDC) estimated that over 96 million U.S. adults had prediabetes, accounting for more than one in three adults (Boltri et al., 2023).

Anthropometric indices are measurements that assess body size, shape, and composition. Traditional indices include Body Mass Index (BMI) and waist circumference, which have been widely used to evaluate obesity and related health risks (Liu et al., 2021). However, newer indices have been developed to provide more accurate assessments of health risks associated with body fat distribution. One such index is the Body Roundness Index (BRI), which incorporates waist and hip measurements to calculate body roundness. Recent studies suggest that BRI may be a better predictor of health outcomes, including the risk of developing prediabetes and diabetes, compared to traditional measures like BMI (Qiu et al., 2024; Wang et al., 2025). The conicity index measures the degree of abdominal adiposity by considering weight, height, and waist circumference; elevated conicity index values are associated with higher diabetes risk (Andrade et al., 2016). The abdominal volume index (AVI) estimates abdominal fat volume using waist and hip measurements and has been linked to metabolic syndrome components, including insulin resistance (Ramírez-Manent et al., 2023). The body adiposity index (BAI), which calculates body fat percentage using hip circumference and height, has shown correlations with diabetes risk factors, though its predictive power of adiposity may be less robust compared to other indices (Freedman et al., 2012). However, a study found that BAI was a better risk predictor of diabetes than BMI and Waist circumference (Alvim et al., 2014). Lastly, A Body Shape Index (ABSI) incorporates waist circumference adjusted for height and weight; while ABSI has been proposed as a predictor of mortality, its association with diabetes risk is less clear compared to other measures (Shafran et al., 2024).

Some studies have proposed novel anthropometric measures in predicting prediabetes in various populations (Klisić et al., 2023; Nayak et al., 2018; Qiu et al., 2024). While several studies have utilized the National Health and Nutrition Examination Survey (NHANES) data to explore the association between individual anthropometric measures and prediabetes risk, a comprehensive analysis comparing the predictive power of multiple anthropometric indices within this dataset remains limited. For instance, research has examined the relationship between the BRI and prediabetes prevalence (Qiu et al., 2024), as well as the association between the Visceral Adiposity Index (VAI) and prediabetes risk (Zheng et al., 2024). However, no study has systematically evaluated and compared the predictive performance of a wide range of anthropometric measures within the NHANES population. By conducting such a comparative analysis, this study provides new insights into which indices are most informative for identifying adults at risk for prediabetes.

Unlike previous studies that focused on individual anthropometric indices, our study provides a comprehensive, head-to-head comparison of nine traditional and novel measures (BMI, Waist circumference, WHR, WHtR, conicity index, AVI, BRI, BAI, ABSI) in the most recent 2021–2023 NHANES cycles, allowing direct assessment of their relative performance in identifying adults at risk for prediabetes. Specifically, we aimed to evaluate the associations between these indices and prediabetes risk and to determine their diagnostic performance in identifying individuals at elevated risk.

## 2 Material and methods

### 2.1 Study design and population

This cross-sectional study utilized data from NHANES, a program designed to assess the health and nutritional status of adults and children in the United States through interviews and physical examinations. NHANES employs a complex, multistage probability sampling method to ensure a representative sample of the U.S population (https://wwwn.cdc.gov/nchs/nhanes/analyticguidelines.aspx). The Ethics Review Board of the National Center for Health Statistics population approved all study protocols, and written informed consents were provided to all participants.

Participants aged 20 years and older from the NHANES cycles spanning 2021 to 2023 were included in the analysis. Individuals with missing data on key variables, such as anthropometric measurements or laboratory results pertinent to prediabetes diagnosis, were excluded. Pregnant women were also excluded due to physiological changes affecting anthropometric and metabolic parameters. The total number of participants included in the analysis was 2515, with a mean age of 48.02 (47.32, 48.72) years. Of these, 48.2 % were males and 51.8 % were females. The majority of participants (64.8 %) were identified as non-Hispanic white individuals, 11.2 % were non-Hispanic Black, 18.1 % were Hispanic, and 5.8 % were non-Hispanic Asian. Overall, 49.6 % of the participants were categorized as having prediabetes (Table 1).

**Table 1:** Characteristics of study participants by prediabetes status among U.S. adults: data from National Health and Nutrition Examination Survey, 2021–2023

Variable	Non-prediabetes	Prediabetes	*P*-value
**Gender** Male Female	377 (36.1) 668 (63.9)	709 (48.2) 761 (51.8)	<0.01
**Age group** 20–44 years 45–64 years 64+ years	543 (52) 302 (28.9) 200 (19.1)	356 (24.2) 542 (36.9) 572 (38.9)	<0.01
**Plasma fasting glucose, (mg/dL)**	92.33 (5.32)	105.17 (8.12)	<0.01
**Glycosylated hemoglobin, %**	5.2 (0.29)	5.61 (0.37)	<0.01
**Race** Hispanic Non- Hispanic white Non- Hispanic Black Non- Hispanic Asian	176 (18.1) 654 (67.1) 87 (8.9) 58 (5.9)	252 (18.1) 900 (64.8) 156 (11.2) 81 (5.8)	0.32
**Education level** < high school High school College graduate ≤	84 (8) 184 (17.6) 777 (74.4)	169 (11.5) 310 (21.1) 991 (67.4)	<0.01
**Marital status** Married Widowed/Divorced/Separated Never married	587 (56.3) 193 (18.5) 263 (25.2)	877 (59.7) 361 (24.6) 230 (15.7)	<0.01
**Income (%FIPR)** <130 %130 % –349 % ≥350 %	183 (19.9) 311 (33.9) 424 (46.2)	239 (18.6) 473 (36.8) 575 (44.7)	0.36
**Anthropometric measures** Weight (kg) BMI (kg/m^2^) waist circumference (cm) WHR WtHR CI AVI BRI BAI ABSI	76.87 (19.07) 27.48 (6.2) 93.77 (14.97) 0.89 (0.08) 0.56 (0.09) 1.27 (0.08) 18.17 (5.96) 4.76 (2.06) 30.88 (7.1) 0.08 (0.01)	84.45 (21.05) 30.15 (6.97) 102.7 (15.37) 0.94 (0.07) 0.61 (0.09) 1.33 (0.08) 21.64 (6.63) 5.95 (2.31) 32.47 (8.1) 0.082 (0.01)	<0.01 <0.01 <0.01 <0.01 <0.01 <0.01 <0.01 <0.01 <0.01 <0.01

Continuous variables are expressed by means and standard deviation (SD), categorical variables are expressed in numbers (percentages). Abbreviations: FIPR, family income to poverty level ratio; BMI, Body Mass Index; ABSI, A body shape index; WHR, Waist-to-hip ratio; WHtR, Waist to height ratio; CI, Conicity index; AVI, Abdominal volume index; BRI, Body roundness index; BAI, Body adiposity index

### 2.2 Measures

**Fig. 1:**
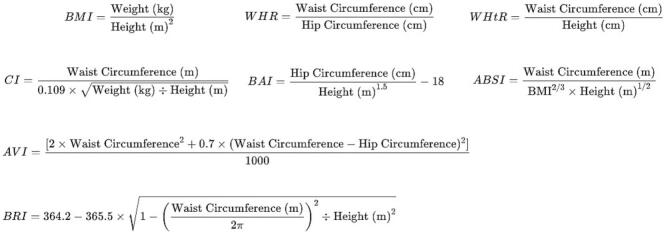
Formulas to calculate the anthropometric measures among U.S. adults: data from National Health and Nutrition Examination Survey, 2021–2023.

Anthropometric data were collected by trained health technicians following standardized protocols. Measurements included weight, height, waist circumference, and hip circumference. These measurements were used to calculate various indices (Fig. 1). Prediabetes based on the 2020 American Diabetes Association's criteria were defined as: (1) having a fasting plasma glucose level between 100 and 125 mg/dL (5.6–6.9 mmol/L) only, (2) HbA1c level between 5.7 % and 6.4 % only, or (3) both impaired fasting glucose and HbA1c level between 5.7 % and 6.4 % (Committee, 2024).

In this study, we included several covariates to control for potential confounding factors. Age was treated as a continuous variable, representing the participant's age in years at the time of the interview. Sex was categorized as male or female. Race/ethnicity was classified into four groups: non-Hispanic White, non-Hispanic Black, Hispanic, and non-Hispanic Asian. Education levels were classified into three categories: less than high school, high school graduates, and college graduates and above. Marital status was defined as married/living with partner, never married, or previously married (including widowed, divorced, or separated).

### 2.3 Statistical analysis

All analyses accounted for the complex survey design of NHANES by incorporating sample weights, strata, and primary sampling units, as recommended in the NHANES Analytic Guidelines (https://wwwn.cdc.gov/nchs/nhanes/analyticguidelines.aspx). Descriptive statistics were used to summarize participant characteristics stratified by prediabetes status. Continuous variables were reported as means and standard deviations (SD) and compared using independent sample *t*-tests. Categorical variables were presented as frequencies and percentages and compared using chi-square tests. Multivariable logistic regression models were employed to assess the associations between quartiles of each anthropometric index and the odds of prediabetes, The models were adjusted for potential confounders, including age, sex, race, socioeconomic status (education and income), physical activity level, and marital status. Adjusted odds ratios (aORs) with 95 % confidence intervals (CIs) were reported. Receiver Operating Characteristic (ROC) curve analysis was conducted to evaluate the discriminatory power of each anthropometric index for identifying prediabetes. The area under the ROC curve (AUC) was calculated for each index, and comparisons between AUCs were made using DeLong's test (DeLong et al., 1988; Shaffer, 1995). The ability of each anthropometric index to predict prediabetes was shown as areas under the ROC curves and the CIs. Optimal cutoff points were determined based on the Youden index, and corresponding sensitivity and specificity values were reported. Higher sensitivity indicates better identification of at-risk individuals, while higher specificity reflects accurate exclusion of low-risk individuals. Missing values (demographics, anthropometric indices, and clinical covariates) were addressed using multiple imputations by Fully Conditional Specification (FCS) with 10 repeated imputed datasets to account for uncertainty and minimize bias. All statistical analyses were performed using SPSS Statistics for Windows, version 25.0 (SPSS Inc., Chicago, Ill., USA), with a significance level set at *P* < 0.05.

## 3 Results

### 3.1 Association between anthropometric measures and risk of prediabetes

The associations between various measured anthropometric indices and the risk of prediabetes in are demonstrated in Table 2. Participants in the highest quartile of BMI (≥32.4 kg/m^2^) had a markedly increased risk of prediabetes, with aOR of 3.71 (95 % CI; 2.82, 4.88). Similarly, those in the highest quartile of waist circumference (≥108.6 cm) had 4.07 times greater odds of prediabetes (95 % CI; 3.08, 5.38). A higher WHR was significantly associated with prediabetes, with participants in the highest quartile of WHR (≥0.98) showing an aOR of 4.16 (95 % CI; 3.01, 5.75). The WHtR also showed an association, with participants in the highest quartile of WHtR (≥0.65) having an aOR of 3.98 (95 % CI; 3.0, 5.26). The conicity index was correlated with prediabetes risk, with participants in the highest quartile of conicity index (≥1.37) having an aOR of 4.71 (95 % CI; 3.46, 6.4). For AVI, participants in the highest quartile of AVI (≥23.63) had significantly increased odds of prediabetes (aOR: 3.99, 95 % CI; 3.02, 5.26). The BRI also showed a positive association with prediabetes, with an aOR of 3.99 (95 % CI; 3.01, 5.26) for participants in the highest quartile of BRI (≥6.64). Participants in the highest quartile of BAI (≥35.96) had an aOR of 3.74 (95 % CI; 2.69, 5.2) for prediabetes. Furthermore, a higher ABSI was linked to an increased risk of prediabetes, with participants in the highest quartile of ABSI (≥0.08) showing an aOR of 2.51 (95 % CI; 1.84, 3.43).

**Table 2:** Associations of anthropometric indices with prediabetes among U.S. adults: data from National Health and Nutrition Examination Survey, 2021–2023

**Variable**	**Unadjusted OR (95 %CI)**	**Model 1 OR (95 % CI)**	**Model 2 OR (95 % CI)**
**BMI** Q1 (<24.3) Q2 (24.3 to <27.8) Q3 (27.8 to <32.4) Q4 (32.4≤)	1 1.78 (1.43, 2.23) 2.53 (2.01, 3.17) 3.42 (2.7, 4.31)	1 1.65 (1.29, 2.1) 2.32 (1.81, 2.97) 3.71 (2.88, 4.79)	1 1.73 (1.33, 2.25) 2.24 (1.71, 2.93) 3.71 (2.82, 4.88)
**Waist circumference** Q1 (<87.5) Q2 (87.5 to <97.6) Q3 (97.6 to <108.6) Q4 (108.6≤)	1 2.5 (2, 3.14) 3.84 (3.04, 4.85) 5.4 (4.25, 6.87)	1 2.07 (1.62, 2.64) 2.93 (2.27, 3.78) 4.34 (3.35, 5.62)	1 1.93 (1.49, 2.52) 2.75 (2.09, 3.61) 4.07 (3.08, 5.38)
**WHR** Q1 (<0.866) Q2 (0.866 to <0.925) Q3 (0.925 to <0.983) Q4 (0.983≤)	1 2.02 (1.62, 2.53) 3.87 (3.06, 4.89) 6.49 (5.06, 8.33)	1 1.78 (1.4, 2.26) 3.1 (2.39, 4.03) 4.6 (3.4, 6.21)	1 1.67 (1.29, 2.16) 2.75 (2.08, 3.62) 4.16 (3.01, 5.75)
**WtHR** Q1 (<0.523) Q2 (0.523 to <0.585) Q3 (0.585 to <0.651) Q4 (0.651≤)	1 2.21 (1.78, 2.77) 4.39 (3.48, 5.55) 4.48 (3.54, 5.68)	1 1.99 (1.56, 2.53) 3.54 (2.76, 4.55) 4.15 (3.2, 5.38)	1 2.01 (1.55, 2.61) 3.31 (2.52, 4.33) 3.98 (3, 5.26)
**Conicity index** Q1 (<1.24) Q2 (1.24 to <1.31) Q3 (1.31 to <1.37) Q4 (1.37≤)	1 2.65 (2.13, 3.1) 3.52 (2.78, 4.45) 7.57 (5.84, 9.8)	1 2.09 (1.65, 2.64) 2.39 (1.86, 3.09) 4.76 (3.58, 6.33)	1 2.09 (1.62, 2.69) 2.16 (1.64, 2.85) 4.71 (3.46, 6.4)
**AVI** Q1 (<15.41) Q2 (15.41 to <19.17) Q3 (19.17 to <23.63) Q4 (23.63≤)	1 2.39 (1.91, 2.99) 3.7 (2.93, 4.68) 5.25 (4.13, 6.68)	1 1.98 (1.55, 2.52) 2.86 (2.22, 3.69) 4.24 (3.28, 5.5)	1 1.84 (1.41, 2.4) 2.7 (2.05, 3.54) 3.99 (3.02, 5.26)
**BRI** Q1 (<3.8) Q2 (3.8 to <5.1) Q3 (5.1 to <6.64) Q4 (6.64≤)	1 2.24 (1.79, 2.79) 4.41 (3.49, 5.57) 4.5 (3.51, 5.7)	1 2 (1.58, 2.55) 3.56 (2.77, 4.57) 4.16 (3.21, 5.4)	1 2.03 (1.56, 2.63) 3.32 (2.53, 4.35) 3.99 (3.01, 5.26)
**BAI** Q1 (<26.17) Q2 (26.17 to <30.27) Q3 (30.27 to <35.96) Q4 (35.96≤)	1 1.42 (1.14, 1.76) 1.55 (1.25, 1.94) 1.71 (1.37, 2.15)	1 1.88 (1.47, 2.41) 2.87 (2.17, 3.79) 3.84 (2.83, 5.21)	1 1.91 (1.47, 2.49) 2.95 (2.19, 3.99) 3.74 (2.69, 5.2)
**ABSI** Q1 (<0.078) Q2 (0.078 to <0.081) Q3 (0.081 to <0.085) Q4 (0.085≤)	1 2.33 (1.86, 2.93) 2.86 (2.3, 3.56) 4.97 (3.86, 6.41)	1 1.85 (1.45, 2.35) 2.01 (1.58, 2.55) 2.52 (1.89, 3.36)	1 1.87 (1.43, 2.43) 1.96 (1.51, 2.53) 2.51 (1.84, 3.43)

Abbreviations: BMI, Body Mass Index; ABSI, A body shape index; WHR, Waist-to-hip ratio; WHtR, Waist to height ratio; AVI, Abdominal volume index; BRI, Body roundness index; BAI, Body adiposity index. Model^1^: crude model. Model ^2^: adjusted for age, sex, race, Physical activity status, household income, marital and education status

### 3.2 Analysis of diagnostic value of anthropometric measures in prediabetes

**Fig. 2:**
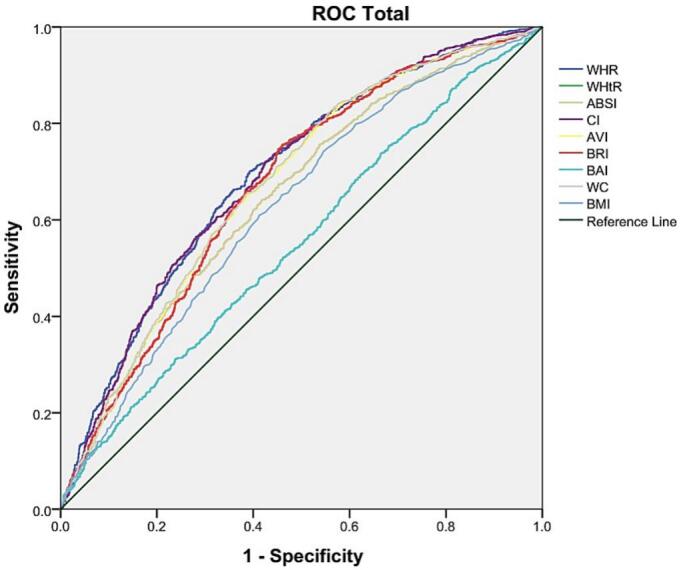
The ROC curve to assess the overall diagnostic performance of anthropometric measures for identifying prediabetes among U.S. adults: data from National Health and Nutrition Examination Survey, 2021–2023.

The ROC curves for identifying participants with diabetes are presented in Fig. 2. Among the nine anthropometric parameters, the WHR demonstrated the highest discrimination ability, with an AUC of 0.69 (95 % CI; 0.67, 0.71). The optimal cutoff value of WHR, determined using the highest Youden index, was 0.90, yielding a sensitivity of 0.70 and a specificity of 0.60. The conicity index also showed an association with an AUC of 0.69 (95 % CI; 0.67, 0.71), sensitivity of 0.73, and specificity of 0.55 at the optimal cutoff of 1.28. The AUCs for the other anthropometric indices were as follows: waist circumference (0.67, 95 % CI; 0.65, 0.69), WHtR and BRI both had an AUC of 0.670 (95 % CI; 0.64, 0.69), and AVI (0.67; 95 % CI: 0.65, 0.69). In contrast, BMI showed lower discrimination ability, with an AUC of 0.626 (95 % CI; 0.60, 0.64), followed by ABSI, (0.64; 95 % CI; 0.62, 0.67). BAI, however, had the lowest AUC of 0.55 (95 % CI; 0.52, 0.57), indicating limited diagnostic value (Table 3). Separate ROC curve analyses conducted for male and female subgroups revealed similar trends (Table 4). In females, WHR demonstrated the best discrimination power, with an AUC of 0.70 (95 % CI; 0.67, 0.72). In males, conicity index exhibited the highest AUC value at 0.67 (95 % CI; 0.63, 0.70).

**Table 3:** Comparison of anthropometric indices in diagnosing prediabetes among U.S. adults: data from National Health and Nutrition Examination Survey, 2021–2023

**Test**	**AUC**	**95 % CI**	**Cutoff**	**Sensitivity**	**Specificity**
BMI	0.62	0.60, 0.64	27.45	0.61	0.58
Waist circumference	0.67	0.65, 0.69	95.95	0.65	0.61
WHR	0.69	0.67, 0.71	0.90	0.70	0.60
WtHR	0.67	0.64, 0.69	0.55	0.75	0.53
Conicity index	0.69	0.67, 0.71	1.28	0.7	0.55
AVI	0.67	0.65, 0.69	18.53	0.65	0.61
BRI	0.67	0.64, 0.69	4.38	0.75	0.53
BAI	0.55	0.52, 0.57	26.97	0.73	0.33
ABSI	0.64	0.62, 0.67	0.08	0.64	0.58

Abbreviations: AUC, area under the curve; CI, confidence interval; BMI, Body Mass Index; ABSI, A body shape index; WHR, Waist-to-hip ratio; WHtR, Waist to height ratio; AVI, Abdominal volume index; BRI, Body roundness index; BAI, Body adiposity index.

**Table 4:** Comparison of anthropometric indices in diagnosing prediabetes by gender among U.S. adults: data from National Health and Nutrition Examination Survey, 2021–2023

Test		AUC	95 % CI	Cutoff	Sensitivity	Specificity
BMI	Male	0.60	0.57, 0.64	27.65	0.55	0.62
	Female	0.64	0.61, 0.67	25.45	0.76	0.46
Waist circumference	Male	0.64	0.60, 0.67	99.15	0.61	0.64
	Female	0.68	0.65, 0.71	88.45	0.80	0.49
WHR	Male	0.67	0.63, 0.70	0.96	0.63	0.63
	Female	0.70	0.67, 0.72	0.90	0.57	0.74
WHtR	Male	0.65	0.62, 0.69	0.55	0.70	0.57
	Female	0.69	0.66, 0.72	0.55	0.80	0.52
Conicity index	Male	0.67	0.63, 0.70	1.28	0.75	0.53
	Female	0.69	0.67, 0.72	1.28	0.71	0.58
AVI	Male	0.64	0.60, 0.67	19.79	0.60	0.64
	Female	0.68	0.65, 0.71	16.24	0.77	0.52
BRI	Male	0.65	0.62, 0.69	4.38	0.70	0.57
	Female	0.69	0.66, 0.72	4.41	0.80	0.52
BAI	Male	0.61	0.58, 0.65	26.65	0.54	0.65
	Female	0.62	0.59, 0.65	32.12	0.69	0.49
ABSI	Male	0.64	0.61, 0.68	0.08	0.49	0.73
	Female	0.63	0.60, 0.66	0.07	0.77	0.43

Abbreviations: AUC, area under the curve; CI, confidence interval; BMI, Body Mass Index; ABSI, A body shape index; WHR, Waist-to-hip ratio; WHtR, Waist to height ratio; AVI, Abdominal volume index; BRI, Body roundness index; BAI, Body adiposity index.

## 4 Discussion

This study investigates the associations between various anthropometric indices and the risk of prediabetes, utilizing data from the NHANES 2021–2023 cycles. Our findings indicate that all measured anthropometric measures including BMI, waist circumference, WHR, WHtR, conicity index, AVI, BRI, BAI, and ABSI were significantly associated with an increased risk of prediabetes in both adjusted and unadjusted models. Notably, WHR demonstrated the highest discriminatory power among the indices evaluated in total population and females.

Our results align with existing literature emphasizing the importance of central adiposity measures in predicting prediabetes and type 2 diabetes risks. For instance, a study by Sadeghi et al. compared various anthropometric indices among first-degree relatives of diabetic patients and found that measures like waist circumference, WHR, and WHtR were more effective predictors of type 2 diabetes mellitus than BMI in an Iranian population (Sadeghi et al., 2024). Similarly, research by Aghaei et al. highlighted a strong association between WHR and diabetes in Persian cohort, suggesting that WHR may be a superior indicator of diabetes risk compared to BMI (Aghaei et al., 2024). Furthermore, a study by Khader et al. evaluated the performance of various anthropometric measures and concluded that WHtR were better predictors of diabetes mellitus and hypertension among Jordanian adults (Khader et al., 2019). Other NHANES studies examined the association between BRI and VAI with diabetes and found that these central obesity indices were associated with the risk of diabetes and prediabetes (Qiu et al., 2024; Zheng et al., 2024). Liu et al. found that WtHR and conicity index were the best predictors of diabetes in the US population (Liu et al., 2022). Vera-Ponce et al. in a meta-analysis study suggested that BMI is a less effective predictor of prediabetes risk compared to measures like WHtR and waist circumference (Vera-Ponce et al., 2024). A systematic review and meta-analysis by Jayedi et al. highlighted that measures of central obesity, such as waist circumference and WHR, are strongly associated with type 2 diabetes risk independent of overall adiposity (Jayedi et al., 2022).

Our study's findings have clear clinical and public health implications. All evaluated adiposity measures were linked to prediabetes risk, with central-obesity indices (e.g. WHR, conicity index) showing the largest associations. These results align with recent NHANES analyses showing that central or visceral adiposity indices outperform BMI for identifying dysglycemia. For example, (Qiu et al., 2024) reported that the Body Roundness Index was a strong predictor of diabetes and prediabetes in NHANES 2007–2018. (Zheng et al., 2024) reported a positive, nonlinear association between the Visceral Adiposity Index (VAI) and diabetes/prediabetes risk in NHANES analyses and recommended considering VAI in clinical risk management. Other work has shown that lipid-derived indices such as the Lipid Accumulation Product (LAP) and VAI track closely with glycemic risk and may add incremental predictive value beyond anthropometry alone (Ahn et al., 2019). The superior predictive ability of central adiposity measures likely reflects the biological relevance of visceral fat, which is metabolically active and contributes to insulin resistance, chronic low-grade inflammation, and dysregulated lipid and glucose metabolism (Maleki et al., 2021). In contrast, BMI reflects overall body mass without distinguishing fat distribution, and therefore may not capture the specific metabolic risks associated with central fat accumulation.

At the same time, our observation that no single anthropometric index achieved excellent discrimination (all AUCs ≤0.70) mirrors findings from the ILERVAS cohort and prior studies showing modest AUCs for prediabetes detection, indicating that these measures should supplement rather than replace laboratory screening (Sánchez et al., 2021). In practical terms, simple central adiposity measurements such as WHR, conicity index, WHtR, and BRI are low cost, easy to implement at the point of care, and can help clinicians triage patients for early metabolic evaluation (fasting glucose or HbA1c) and provide targeted lifestyle counselling, while recognizing that anthropometry alone cannot definitively diagnose dysglycemia. Incorporating WHR and conicity index into routine clinical screenings could enable earlier identification of high-risk individuals, allowing timely lifestyle interventions, though implementation may require standardized protocols, staff training, and integration into workflow.

The varying performance of anthropometric indices in predicting prediabetes risk highlights the importance of selecting appropriate measures for clinical and public health applications. Central adiposity indices, particularly WHR and conicity index, demonstrated superior predictive capabilities in our study. WHR, which reflects the distribution of fat between the waist and hips, may capture visceral fat accumulation more effectively than BMI, which does not account for fat distribution (Elsayed et al., 2008). Conicity index, which incorporates weight, height, and waist circumference, provides an assessment of abdominal fat distribution and has been shown to be a reliable indicator of cardiovascular and metabolic risk (Martins et al., 2023). In contrast, BMI, a widely used measure of general adiposity, demonstrated a weaker association with prediabetes risk in our study. This may be due to inability of BMI to distinguish between lean and fat mass and its failure to account for fat distribution, which are important factors in metabolic risk (Buss, 2014).

A major strength of this study is the use of a large, nationally representative sample from NHANES, which enhances the generalizability of our findings to the U.S. adult population. Additionally, the comprehensive assessment of multiple anthropometric indices allows for a thorough comparison of their predictive abilities for prediabetes risk. However, several limitations should be acknowledged. The cross-sectional design of the study precludes the establishment of causal relationships between anthropometric measures and prediabetes risk. Longitudinal studies are needed to confirm these associations and to determine the temporal sequence of these relationships. Additionally, while we adjusted for several potential confounders, residual confounding cannot be entirely ruled out. Moreover, some potential confounding factors, including dietary habits, and the use of certain medications (such as corticosteroids and antidepressants), were not included in the present analysis. These factors may influence both body composition and glucose metabolism, and their absence could have led to residual confounding.

## 5 Conclusions

In conclusion, this study demonstrates that central adiposity measures, particularly WHR and conicity index, are more clearly associated with prediabetes risk than BMI. These findings underscore the importance of assessing fat distribution in addition to general adiposity when evaluating metabolic risk. However, no single anthropometric measure serves as a perfect biomarker for detecting individuals with prediabetes. Incorporating measures of central adiposity into routine clinical practice may enhance the early identification of individuals at risk for prediabetes and inform targeted prevention strategies. However, due to the cross-sectional nature of our study, causal relationships between anthropometric indices and prediabetes risk cannot be established. Future longitudinal and interventional studies are warranted to confirm these associations.

## CRediT authorship contribution statement

**Xi Chen:** Writing – original draft, Validation, Software, Conceptualization. **Lijun Yan:** Writing – original draft, Software, Conceptualization. **Jie Yang:** Writing – review & editing, Methodology. **Shufang Yang:** Writing – original draft, Investigation.

## Informed consent statement

The NHANES protocol has undergone a thorough review and approval by the research ethics review board at the National Center for Health Statistics. All participants involved in the study provided written informed consent.

## Ethics statement

The research involving human participants underwent a thorough review and received approval from the Research Ethics Review Board of the National Centre for Health Statistics Research Ethics (NCHS). All patients or participants gave their written informed consent to be part of this study.

## Declaration of competing interest

The authors declare that they have no known competing financial interests or personal relationships that could have appeared to influence the work reported in this paper.

## Data Availability

The original contributions presented in the study are included in the article material, further inquiries can be directed to the corresponding authors.
